# Differences in Non-Anthocyanin Phenolics and Antioxidant Capacity of 27 Red Grapevine Varieties Grown in Northern Portugal

**DOI:** 10.3390/molecules31010011

**Published:** 2025-12-19

**Authors:** Miguel Baltazar, Sandra Pereira, Eliana Monteiro, Vânia Silva, Helena Ferreira, Joana Valente, Fernando Alves, Isaura Castro, Berta Gonçalves

**Affiliations:** 1 Centre for the Research and Technology of Agro-Environmental and Biological Sciences (CITAB), Institute for Innovation, Capacity Building and Sustainability of Agri-Food Production (Inov4Agro), University of Trás-os-Montes e Alto Douro (UTAD), 5000-801 Vila Real, Portugal; mbaltazar@utad.pt (M.B.); sirp@utad.pt (S.P.); elianamonteiro@utad.pt (E.M.); al75372@alunos.utad.pt (V.S.); helenaf@utad.pt (H.F.); icastro@utad.pt (I.C.); 2 Symington Family Estates, Travessa Barão de Forrester 86, 4400-034 Vila Nova de Gaia, Portugal; joana.valente@symington.com (J.V.); fernando.alves@symington.com (F.A.)

**Keywords:** abiotic stress, antioxidant activity, climate change, phenolic compounds, varietal selection, *Vitis vinifera* L.

## Abstract

Climate change imposes significant challenges on vitiviniculture, increasing the need to identify more resilient grapevine varieties. While red grape varieties are known for their high anthocyanin content, other phenolic compounds should also be considered when assessing adaptability to biotic and abiotic stresses. For this, the phenolic composition and antioxidant capacity of 27 red *Vitis vinifera* L. varieties grown in Portugal were studied across two years. Under warmer and drier conditions, most varieties exhibited higher total phenolic content (TPC) and antioxidant activity, with ‘Donzelinho Tinto’ and ‘Zinfandel’ displaying the most pronounced increases. These varieties also had the highest increases in phenolic acids and flavan-3-ols, highlighting how environmental stress modulates secondary metabolites. Varieties such as ‘Aragonez’, ‘Trincadeira’, ‘Touriga Franca’, and ‘Tinta Francisca’, demonstrated stable profiles, indicating a robust response to climatic fluctuation. Correlation analysis revealed strong associations between TPC and antioxidant capacity, highlighting the importance of phenolics in mitigating oxidative stress. By identifying varieties with enhanced phenolic and antioxidant plasticity, the diversity observed in this work offers valuable insights for future varietal selection aimed at mitigating climate change-induced challenges. Overall, this work reinforces the potential of varietal selection to promote sustainable viticulture in regions increasingly impacted by climatic variability.

## 1. Introduction

Viticulture is a major agricultural and socio-economic activity worldwide, particularly in Mediterranean regions where wine production is deeply embedded with the cultural identity and economy frameworks [1,2]. Despite being considered a resilient crop, the grapevine (*Vitis vinifera* L.) is being challenged as the climate shifts toward increasingly extreme conditions. In fact, climate change is rapidly altering environmental conditions through rising temperatures, prolonged droughts, and erratic precipitation patterns, all of which affect grapevine physiology and berry composition, ultimately influencing wine quality [3,4,5]. These shifts are expected to be particularly pronounced in Mediterranean climates, where mean temperatures and reduced rainfall are already surpassing historical averages [6,7]. Thus, as climate change is expected to reshape viticulture, several adaptation strategies have become a key focus of research [8,9,10]. One of these strategies is varietal selection, which focuses on the use of the genetic diversity of the grapevine as a valuable resource for mitigating climate-induced challenges, by exploiting the varying capability of different varieties to withstand abiotic stresses [11,12]. However, the variability among grapevine varieties remains largely understudied, especially when it comes to autochthonous varieties, which could offer superior adaptive potential [12,13,14]. Moreover, even if these varieties do not demonstrate increased stress resilience, increased germplasm diversity in viticulture can decrease the expected losses due to climate change [15].

Environmental extremes intensify the abiotic stress experienced by the grapevine, which can trigger metabolic changes that alter the composition of secondary metabolites in grape berries [16,17]. Among these, phenolic compounds, such as flavonoids, including anthocyanins, flavonols and flavan-3-ols, and non-flavonoids, such as hydroxybenzoic and hydroxycinnamic acids and stilbenes, are extremely important in both plant stress resilience and wine quality [18,19,20]. While anthocyanins dominate the phenolic profile of red grape varieties and are widely studied for their influence on wine coloration, non-anthocyanin flavonols and phenolic acids, also play crucial but often underappreciated roles in grape and wine quality [19,21,22]. These compounds contribute to oxidative stability, mouthfeel, and aroma precursors, influencing sensory characteristics beyond color [20]. Moreover, they are involved in plant stress resilience mechanisms such as reactive oxygen species (ROS) scavenging, cell structural strengthening, stomatal closure and osmotic adjustment, while also functioning as signaling molecules to trigger defense genes against biotic and abiotic stresses [23]. For instance, their accumulation and indirect and direct action can help reduce the damaging effects of excessive sunlight and UV radiation [24,25,26], as well as drought and increased temperatures [27,28,29,30]. Therefore, the biosynthesis and accumulation of phenolic compounds is highly sensitive to environmental conditions [19,29]. Moreover, this response can be genotype-dependent, with different grapevine varieties presenting contrasting behaviors [12,31].

A deeper understanding of genotype-environment interactions is therefore critical for improving the climate resilience of viticulture, and the optimization of viticultural management strategies. Thus, this study aimed to explore the varietal differences of 27 red *Vitis vinifera* L. varieties cultivated in northern Portugal, over two contrasting growing seasons characterized by marked temperature and precipitation differences. For this, we quantified total phenolics, flavonoids, *ortho*-diphenols, assessed antioxidant activity (ABTS•^+^, DPPH, FRAP assays), and performed High-performance liquid chromatography with diode array detection (HPLC-DAD) analysis to elucidate non-anthocyanin phenolic profiles, including hydroxybenzoic acids, hydroxycinnamic acids, flavan-3-ols, and flavonols. By examining the varietal responses to interannual climatic variability, this work aims to elucidate how different grapevine varieties behave under the same growth conditions.

## 2. Results

### 2.1. Climatic Conditions

**Figure 1:**
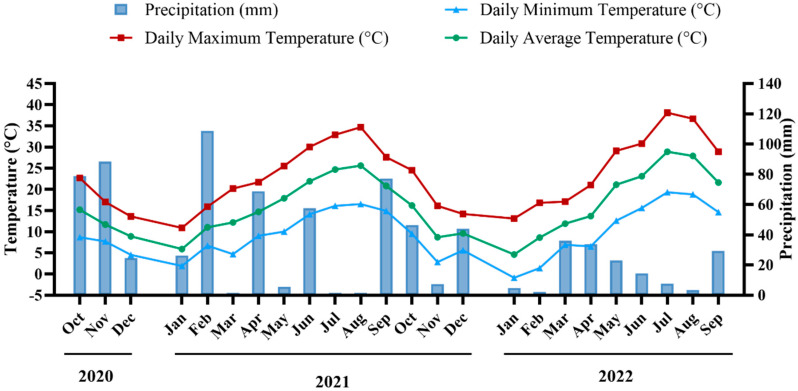
Monthly average values of daily temperature and precipitation in the vineyard from October 2020 to September 2022.

Monthly temperature trends and precipitation levels from October 2020 to September 2022 are represented in Figure 1. Overall, 2022 was a warmer and dryer year compared to 2021. In fact, during the first growing season (October 2020 to September 2021), the mean daily temperature was about 15.9 °C, gradually rising from the cooler autumn and winter months to a peak daily maximum of 34.7 °C in August. This season was marked by substantial precipitation, accumulating roughly 536 mm, occurring especially during the wetter winter period. Contrastingly, the second growing season (October 2021 to September 2022) exhibited a higher mean temperature of around 16.3 °C, with a relatively higher peak daily maximum of 38.1 °C in July. Moreover, this season received significantly less rainfall, totaling only about 252 mm, exacerbating water stress conditions during the critical growing months. Furthermore, between the first and second seasons, the number of days above 35 °C doubled, being 31 days for the period comprised between October 2020 and September 2021, and 62 days for the period between October 2021 and September 2022; once more highlighting the contrasting differences between seasons.

### 2.2. Determination of Total Phenolic Content

**Figure 2:**
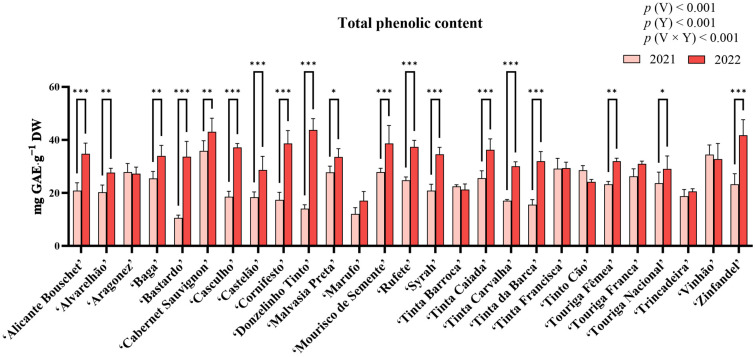
Total phenolic content in the berry extracts of the red varieties under study. Results are expressed as mg GAE·g^−1^ DW. Values presented as mean ± standard deviation. For statistically significant differences between years within varieties: *—*p* < 0.05; **—*p* < 0.01; ***—*p* < 0.001. *p*(V), *p*(Y), and *p*(V × Y) correspond to the *p* values of the main effects of variety, year, and their interaction. V—variety; Y—year.

Figure 2 presents the results from the quantification of total phenolic content (TPC). ANOVA revealed statistically significant differences for variety, year, and their interaction (*p* < 0.001). Variety and year accounted for most of the variation observed (above 30% for both). In 2021, the highest TPC was observed in ‘Vinhão’ (34.53 ± 3.56 mg gallic acid equivalents (GAE)·g^−1^ dry weight (DW)), while the lowest was recorded in ‘Bastardo’ (10.61 ± 1.06 mg GAE·g^−1^ DW). For 2022, ‘Donzelinho Tinto’ showed the highest TPC (43.76 ± 4.30 mg GAE·g^−1^ DW), whereas ‘Tinta Barroca’ had the lowest (21.26 ± 2.14 mg GAE·g^−1^ DW). Statistically significant increases (*p* < 0.05) in TPC from 2021 to 2022 were observed in almost all varieties, with only ‘Aragonez’, ‘Marufo’, ‘Tinta Barroca’, ‘Tinta Francisca’, ‘Tinto Cão’, ‘Touriga Franca’, ‘Trincadeira’, and ‘Vinhão’ presenting no statistically significant differences between years.

### 2.3. Quantification of Ortho-Diphenols

**Figure 3:**
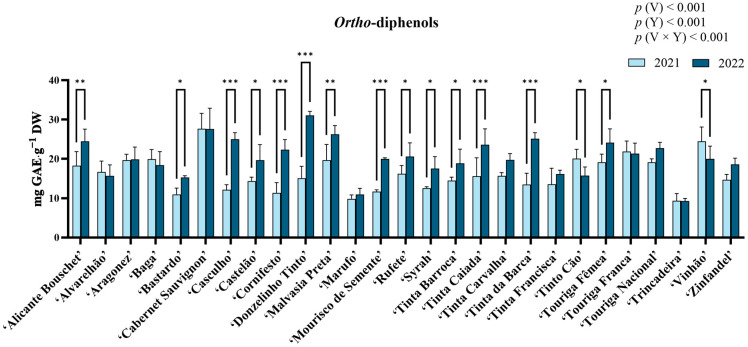
*Ortho*-diphenol content in the berry extracts of the red varieties under study. Results are expressed as mg GAE·g^−1^ DW. Values presented as mean ± standard deviation. For statistically significant differences between years within varieties: *—*p* < 0.05; **—*p* < 0.01; ***—*p* < 0.001. *p*(V), *p*(Y), and *p*(V × Y) correspond to the *p* values of the main effects of variety, year, and their interaction. V—variety; Y—year.

*Ortho*-diphenol quantification results are presented in Figure 3. ANOVA revealed statistically significant differences for variety, year, and their interaction (*p* < 0.05), with variety contributing with the largest proportion of variation (50.79%). In 2021, the highest *ortho*-diphenol content was observed in ‘Vinhão’ (24.49 ± 3.62 mg GAE·g^−1^ DW), while in 2022 it was observed for ‘Donzelinho Tinto’ (31.06 ± 1.05 mg GAE·g^−1^ DW). ‘Trincadeira’ revealed the lowest values in both years (9.35 ± 1.80 mg GAE·g^−1^ DW in 2021 and 9.28 ± 0.67 mg GAE·g^−1^ DW in 2022). Statistically significant increases (*p* < 0.05) in *ortho*-diphenol content between years were observed in most varieties, with only ‘Tinto Cão’ and ‘Vinhão’ decreasing significantly (*p* < 0.05), while ‘Alvarelhão’, ‘Aragonez’, ‘Baga’, ‘Cabernet Sauvignon’, ‘Marufo’, ‘Tinta Carvalha’, ‘Tinta Francisca’, ‘Touriga Franca’, ‘Touriga Nacional’, ‘Trincadeira’, and ‘Zinfandel’ presented no differences between years.

### 2.4. Quantification of Flavonoids

**Figure 4:**
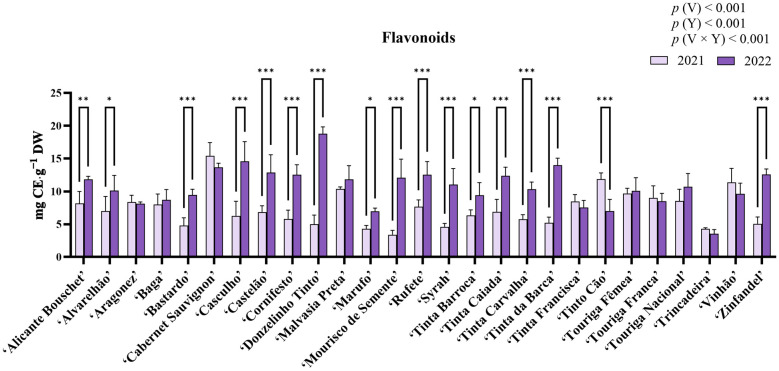
Flavonoid content in the berry extracts of the red varieties under study. Results are expressed as mg CE·g^−1^ DW. Values presented as mean ± standard deviation. For statistically significant differences between years within varieties: *—*p* < 0.05; **—*p* < 0.01; ***—*p* < 0.001. *p*(V), *p*(Y), and *p*(V × Y) correspond to the *p* values of the main effects of variety, year, and their interaction. V—variety; Y—year.

For flavonoid quantification, results are presented in Figure 4, and ANOVA revealed statistically significant differences for variety, year, and the interaction variety × year (*p* < 0.05). For this quantification, variety and variety × year accounted for 30.21%, and 32.55% of the total variation, respectively. The highest flavonoid content in 2021 was observed in ‘Cabernet Sauvignon’ (15.42 ± 2.03 mg catechin equivalents (CE)·g^−1^ DW), while the lowest was in ‘Mourisco de Semente’ (3.41 ± 0.67 mg CE·g^−1^ DW). In 2022, ‘Donzelinho Tinto’ showed the highest value (18.78 ± 1.06 mg CE·g^−1^ DW), whereas ‘Trincadeira’ had the lowest (3.55 ± 0.64 mg CE·g^−1^ DW). Statistically significant increases (*p* < 0.05) in *ortho*-diphenol content between years were observed in most varieties. Nonetheless, ‘Tinto Cão’ presented a statistically significant decrease, while ‘Aragonez’, ‘Baga’, ‘Cabernet Sauvignon’, ‘Malvasia Preta’, ‘Tinta Carvalha’, ‘Tinta Francisca’, ‘Touriga Fêmea’, ‘Touriga Franca’, ‘Touriga Nacional’, and ‘Trincadeira’ revealed no statistically significant differences.

### 2.5. Quantification of Individual Non-Anthocyanin Phenolic Compounds

Results of the quantification of non-anthocyanin phenolic compounds are presented in Table 1 and Table 2. Gallic acid content in the red grape varieties under study ranged from 3.82 ± 1.79 µg·g^−1^ DW in ‘Mourisco-de-Semente’ up to 26.68 ± 12.95 µg·g^−1^ DW in ‘Rufete’ in 2021, whereas in 2022 values varied from 4.41 ± 1.78 µg·g^−1^ DW in ‘Trincadeira’ to 69.96 ± 9.78 µg·g^−1^ DW in ‘Donzelinho Tinto’. Overall, a general increase was observed between years. Moreover, two-way ANOVA revealed that gallic acid content was significantly (*p* < 0.05) influenced by variety, year, and by their interaction, with variety accounting for most of the variation observed (above 40%). Between 2021 and 2022, statistically significant increases (*p* < 0.05) were observed in ‘Alicante Bouschet’, ‘Bastardo’, ‘Casculho’, ‘Cornifesto’, ‘Donzelinho Tinto’, ‘Malvasia Preta’, ‘Marufo’, ‘Mourisco-de-Semente’, ‘Syrah’, ‘Tinta Caiada’, ‘Tinta Carvalha’, ‘Tinta da Barca’, ‘Touriga Fêmea’, and ‘Zinfandel’. The remaining varieties presented no significant differences between years.

Among hydroxycinnamic acids, the highest caftaric acid content in 2021 was found in ‘Tinto Cão’ (43.70 ± 9.85 µg·g^−1^ DW), while the lowest was in ‘Bastardo’ (9.02 ± 0.50 µg·g^−1^ DW). In 2022, the lowest value was quantified in ‘Tinta Barroca’ (14.42 ± 5.57 µg·g^−1^ DW), while the highest was in ‘Donzelinho Tinto’ (30.20 ± 11.47 µg·g-1 DW). Statistical analysis revealed that caftaric acid content was significantly (*p* < 0.05) influenced by variety, year, and their interaction. No general behavior was observed, with the response being mostly varietal dependent. Between years, statistically significant increases (*p* < 0.05) were observed in ‘Bastardo’, ‘Castelão’, ‘Donzelinho Tinto’, ‘Mourisco-de-Semente’, and ‘Touriga Fêmea’, while significant decreases (*p* < 0.05) were detected in ‘Alicante Bouschet’, ‘Aragonez’, ‘Casculho’, ‘Tinta Barroca’, and ‘Tinto Cão’. No significant differences were observed for the remaining varieties. Caffeic acid content increased for most varieties in 2022. Values ranged from 8.83 ± 0.16 µg·g^−1^ DW in ‘Bastardo’ up to 22.18 ± 2.45 µg·g^−1^ DW in ‘Aragonez’ in 2021, while in 2022 the highest content was 157.06 ± 36.56 µg·g^−1^ DW in ‘Donzelinho Tinto’ and the lowest 11.11 ± 2.13 µg·g^−1^ DW in ‘Tinta Francisca’. ANOVA revealed that caffeic acid content was significantly influenced by variety, year, and their interaction variety × year (*p* < 0.05). Specifically, statistically significant increases (*p* < 0.05) were observed in ‘Alicante Bouschet’, ‘Bastardo’, ‘Cabernet Sauvignon’, ‘Casculho’, ‘Castelão’, ‘Donzelinho Tinto’, ‘Rufete’, ‘Syrah’, ‘Tinta Carvalha’, and ‘Zinfandel’. On the other hand, varieties ‘Alvarelhão’, ‘Aragonez’, ‘Baga’, ‘Cornifesto’, ‘Malvasia Preta’, ‘Marufo’, ‘Mourisco-de-Semente’, ‘Tinta Barroca’, ‘Tinta Caiada’, ‘Tinta da Barca’, ‘Tinta Francisca’, ‘Tinto Cão’, ‘Touriga Fêmea’, ‘Touriga Franca’, ‘Touriga Nacional’, ‘Trincadeira’, and ‘Vinhão’ displayed no statistically significant differences between the two growing seasons.

For flavan-3-ols, catechin concentration in 2021 ranged from 14.43 ± 3.09 µg·g^−1^ DW in ‘Bastardo’ to 228.47 ± 60.77 µg·g^−1^ DW in ‘Tinto Cão’, while in 2022 the content varied between 47.75 ± 34.37 µg·g^−1^ DW in ‘Tinta Barroca’ and 165.20 ± 90.35 µg·g^−1^ DW in ‘Cabernet Sauvignon’. Statistical analysis revealed that the interaction, variety, and year all significantly (*p* < 0.05) influenced catechin accumulation, with the interaction accounting for most of the variability (38.50%). Notably, statistically significant increases (*p* < 0.05) from 2021 to 2022 were observed in ‘Aragonez’, ‘Casculho’, ‘Tinta Barroca’, and ‘Tinto Cão’. Varieties ‘Bastardo’, ‘Cabernet Sauvignon’, ‘Castelão’, ‘Donzelinho Tinto’, ‘Mourisco-de-Semente’, and ‘Touriga Fêmea’ decreased significantly (*p* < 0.05) between both growing seasons.

For epicatechin, in 2021 the highest concentration was found in ‘Cabernet Sauvignon’ (404.29 ± 147.61 µg·g^−1^ DW) while the lowest was in ‘Trincadeira’ (33.31 ± 20.37 µg·g^−1^ DW). For 2022, the highest amount of epicatechin was observed in ‘Donzelinho Tinto’ (928.04 ± 225.60 µg·g^−1^ DW), with the lowest being observed in ‘Aragonez’ (41.75 ± 7.32 µg·g^−1^ DW). Statistical analysis indicated that the variety, year and the interaction between variety and year significantly influenced epicatechin content (*p* < 0.05), with variety accounting for 47.47% of the variation observed. Between years, statistically significant increases (*p* < 0.05) were observed in ‘Alicante Bouschet’, ‘Bastardo’, ‘Casculho’, ‘Castelão’, ‘Donzelinho Tinto’, ‘Syrah’, ‘Tinta da Barca’, and ‘Zinfandel’. The only statistically significant decrease (*p* < 0.05) was observed in ‘Tinto Cão’, while the changes in the remaining varieties were not statistically significant.

Quantification of identified flavonols is presented in Table 2. In all varieties, quercetin derivates were the most common flavonol. For myricetin-*O*-glucoside, the lowest amounts in both years were quantified in ‘Bastardo’ (57.85 ± 22.39 and 64.17 ± 21.70 µg·g^−1^ DW respectively), while the highest was found in ‘Vinhão’ in 2021 (564.79 ± 71.32 µg·g^−1^ DW) and ‘Zinfandel’ in 2022 (586.41 ± 238.93 µg·g^−1^ DW). Two-way ANOVA indicated statistically significant effects (*p* < 0.05) for year, variety and their interaction, with variety being the factor influencing myricetin-3-*O*-glucoside accumulation the (61.06). Between 2021 and 2022, statistically significant decreases (*p* < 0.05) were observed in ‘Alicante Bouschet’, ‘Casculho’, and ‘Zinfandel’. On the other hand, ‘Malvasia Preta’, and ‘Vinhão’ were the only varieties with statistically significant increases (*p* < 0.05), while the remained revealed no significant changes in myricetin-3-O-glucoside content. For quercetin-3-o-galactoside, in 2021 values ranged from 15.13 ± 0.47 µg·g^−1^ DW in ‘Trincadeira’ to 88.51 ± 24.80 µg·g^−1^ DW in ‘Vinhão’, while in 2022 the values spanned from undetectable (in ‘Trincadeira’ for example) to 64.30 ± 31.98 µg·g^−1^ DW in ‘Tinta da Barca’. Although the overall year effect was not significant, ANOVA revealed that both variety and the interaction between variety and year had significant effects (*p* < 0.05) on quercetin-3-*O*-galactoside content. Moreover, variety accounted for most of the variation (44.31%). Between growing seasons, significant decreases (*p* < 0.05) were observed in ‘Donzelinho Tinto’ and ‘Tinta Carvalha’, whereas significant increases (*p* < 0.05) were observed in ‘Tinto Cão’, ‘Touriga Franca’, and ‘Vinhão’. Quercetin-3-*O*-glucuronide quantification revealed the content of this compound varied considerably among the red grape varieties. In 2021 values ranged from 57.05 ± 14.17 µg·g^−1^ DW in ‘Tinto Cão’ to 590.90 ± 223.65 µg·g^−1^ DW in ‘Zinfandel’, and in 2022 ranged from 99.71 µg·g^−1^ DW in ‘Trincadeira’ to 709.51 ± 330.16 µg·g^−1^ DW in ‘Zinfandel’. Statistical analysis indicated that both variety (accounting for 54.41% of the total variation) and the year factors significantly (*p* < 0.05) influenced quercetin-3-*O*-glucuronide content, while the interaction between variety and year was not significant. In general, varieties experienced an increase in this compound from 2021 to 2022, being statistically significant (*p* < 0.05) in ‘Alicante Bouschet’, ‘Aragonez’, ‘Cabernet Sauvignon’, ‘Casculho’, ‘Tinta Barroca’, ‘Tinta Carvalha’, and ‘Tinto Cão’. Results of the quercetin-3-*O*-glucoside quantification revealed the content ranged from 40.44 ± 13.38 µg·g^−1^ DW in ‘Cornifesto’ to 244.07 ± 119.12 µg·g^−1^ DW in ‘Touriga Franca’, while in 2022 the values ranged from undetected (below LOQ in ‘Trincadeira’) up to 449.73 ± 143.29 µg·g^−1^ DW in ‘Tinta Caiada’. Two-way ANOVA revealed statistically significant (*p* < 0.05) effects of variety and year on quercetin-3-*O*-glucoside content, with variety accounting for 33.1% of the total variation observed. Statistically significant decreases (*p* < 0.05) were detected in ‘Alicante Bouschet’, ‘Aragonez’, ‘Tinta Barroca’, ‘Tinta Caiada’, ‘Tinta Carvalha’, and ‘Tinto Cão’. On the other hand, significant increases (*p* < 0.05) were noted in ‘Donzelinho Tinto’, ‘Cornifesto’, ‘Touriga Franca’, and ‘Vinhão’. Finally, for isohamnetin-3-*O*-glucoside, in 2021 and 2022 the lowest values were undetectable (below LOQ in ‘Tinta Caiada’ and ‘Trincadeira’), while the highest were 184.90 ± 35.14 µg·g^−1^ DW and 219.54 ± 89.67 µg·g^−1^ DW in ‘Zinfandel’ in 2021 and 2022, respectively. ANOVA revealed that both variety and year significantly (*p* < 0.05) influenced isohamnetin-3-*O*-glucoside concentration, while the interaction effect was not significant. The variety factor accounted for 67.78% of the variability observed. Between the two years, statistically significant decreases (*p* < 0.05) in ‘Aragonez’, ‘Cabernet Sauvignon’, ‘Casculho’, ‘Tinta Barroca’, and ‘Tinta Caiada’.

### 2.6. Analysis of Antioxidant Activity Through the ABTS•^+^ Assay

**Figure 5:**
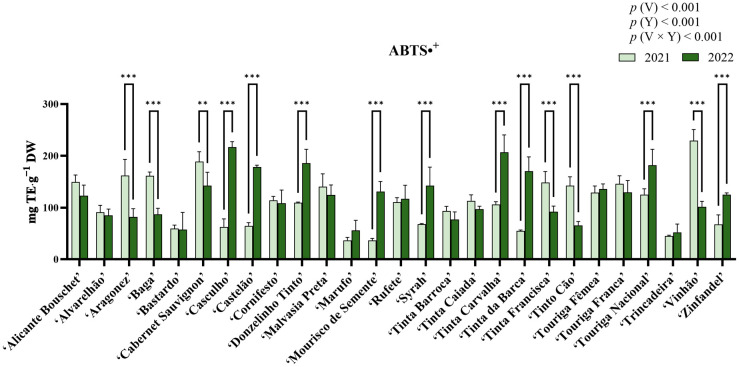
Antioxidant activity measured using the ABTS^•+^ radical scavenging-assay in the berry extracts of the red varieties under study. Results are expressed as mg TE·g^−1^ DW. Values presented as mean ± standard deviation; For statistically significant differences between years within varieties: **—*p* < 0.01; ***—*p* < 0.001. *p*(V), *p*(Y), and *p*(V × Y) correspond to the *p* values of the main effects of variety, year, and their interaction. V—variety; Y—year.

In the analysis of antioxidant activity through the ABTS•^+^ (2,2′-azino-bis (3-ethylbenzothiazoline-6-sulphonic acid)) radical scavenging assay (Figure 5), statistical analysis revealed significant differences for the factors variety, year, and their interaction (*p* < 0.05), with variety contributing the largest proportion of variation (41.69%). For this assay, the highest antioxidant activity for 2021 was observed in ‘Vinhão’ (229.15 ± 21.89 mg Trolox equivalents (TE)·g^−1^ DW), while the lowest was recorded in ‘Marufo’ (36.73 ± 5.726 mg TE·g^−1^ DW). For 2022, ‘Tinta Carvalha’ showed the highest antioxidant activity (207.12 ± 33.37 mg TE·g^−1^ DW), whereas ‘Bastardo’ had the lowest (57.45 ± 33.22 mg TE·g^−1^ DW). Statistically significant increases were observed in ‘Casculho’, ‘Castelão’, ‘Donzelinho Tinto’, ‘Mourisco de Semente’, ‘Syrah’, ‘Tinta Carvalha’, ‘Tinta da Barca’, ‘Touriga Nacional’, and ‘Zinfandel’. Contrastingly, statistically significant decreases in antioxidant activity were observed in ‘Aragonez’, ‘Baga’, ‘Cabernet Sauvignon’, ‘Tinta Francisca’, ‘Tinto Cão’, and ‘Vinhão’. The remaining varieties revealed no significant differences.

### 2.7. Analysis of Antioxidant Activity Through the DPPH Assay

**Figure 6:**
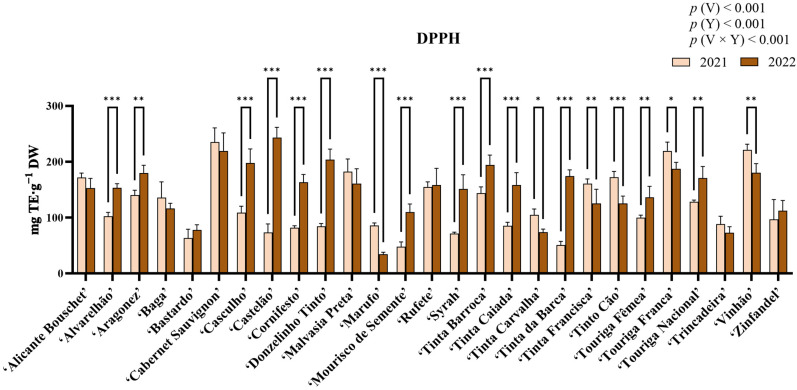
Antioxidant activity measured using the DPPH radical scavenging-assay in the berry extracts of the red varieties under study. Results are expressed as mg TE·g^−1^ DW. Values presented as mean ± standard deviation; For statistically significant differences between years within varieties: *—*p* < 0.05; **—*p* < 0.01; ***—*p* < 0.001. *p*(V), *p*(Y), and *p*(V × Y) correspond to the *p* values of the main effects of variety, year, and their interaction. V—variety; Y—year.

Results from the DPPH (2,2-Diphenyl-1-picrylhydrazyl) radical scavenging assay are shown in Figure 6. Statistical analysis indicated significant differences among variety, year, and their interaction (*p* < 0.05). Among these factors, variety contributed the most to the observed variation (58.34%). In 2021, ‘Cabernet Sauvignon’ exhibited the highest antioxidant activity (235.14 ± 25.56 mg TE·g^−1^ DW), whereas ‘Mourisco de Semente’ recorded the lowest (47.68 ± 8.94 mg TE·g^−1^ DW). In contrast, in 2022, ‘Castelão’ had the highest activity (243.39 ± 18.41 mg TE·g^−1^ DW), while ‘Marufo’ displayed the lowest (34.29 ± 3.70 mg TE·g^−1^ DW). A significant increase in antioxidant activity was observed for several varieties, including ‘Alvarelhão’, ‘Aragonez’, ‘Casculho’, ‘Castelão’, ‘Cornifesto’, ‘Donzelinho Tinto’, ‘Mourisco de Semente’, ‘Tinta Barroca’, ‘Tinta Caiada’, ‘Tinta da Barca’, ‘Touriga Fêmea’, ‘Touriga Nacional’, and ‘Vinhão’. Antioxidant activity declined significantly between 2021 and 2022 for ‘Marufo’, ‘Tinta Carvalha’, ‘Tinta Francisca’, ‘Tinto Cão’, and ‘Touriga Franca’. The remaining varieties revealed no significant differences between years.

### 2.8. Analysis of Antioxidant Activity Through the FRAP Assay

**Figure 7:**
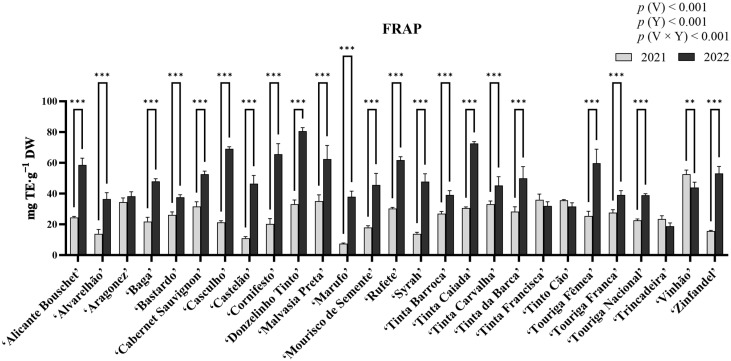
Antioxidant activity measured using FRAP-assay in the berry extracts of the red varieties under study. Results are expressed as mg TE·g^−1^ DW. Values presented as mean ± standard deviation. For statistically significant differences between years within varieties: **—*p* < 0.01; ***—*p* < 0.001. *p*(V), *p*(Y), and *p*(V × Y) correspond to the *p* values of the main effects of variety, year, and their interaction. V—variety; Y—year.

Results from the Ferric Reducing Antioxidant Power (FRAP) assay are presented in Figure 7. Statistical analysis revealed significant differences for variety, year, and the interaction variety × year (*p* < 0.05), with the year factor contributing with the largest proportion of variation (47.89%). Among varieties, in 2021 the highest antioxidant activity was observed in ‘Vinhão’ (52.57 ± 2.73 mg TE·g^−1^ DW), while the lowest was in ‘Marufo’ (7.49 ± 0.40 mg TE·g^−1^ DW). On the other hand, ‘Donzelinho Tinto’ showed the highest antioxidant activity in 2022 (80.61 ± 2.23 mg TE·g^−1^ DW), whereas ‘Trincadeira’ had the lowest (18.73 ± 2.24 mg TE·g^−1^ DW). Statistical analysis revealed that most varieties increased significantly (*p* < 0.05) in antioxidant activity between 2021 and 2022, with only ‘Vinhão’ presenting a significant decrease, and varieties ‘Aragonez’, ‘Tinta Francisca’, ‘Tinto Cão’, and ‘Trincadeira’ presenting no differences.

### 2.9. Pearson Correlation Analysis

**Figure 8:**
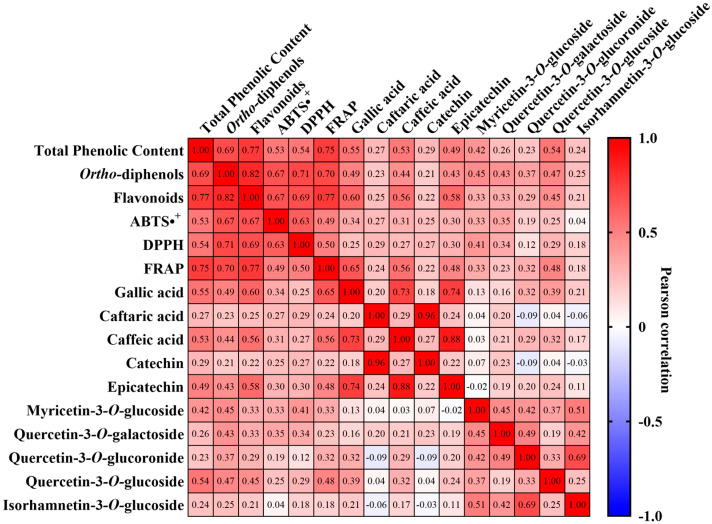
Pearson’s correlation matrix of the phenolic content and antioxidant activity for a confidence level of 5%. Color scale ranges from red (1) to blue (−1).

To better assess the relationships between phenolic content and antioxidant activity, a Pearson correlation analysis was performed (Figure 8). In general, most of correlations observed were positive, indicating these compounds increase together in a general manner, and are also correlated with the antioxidant activity. In fact, total phenolic content (TPC) exhibited statistically significant correlations (*p* < 0.05) with all variables, specifically positive correlations with *ortho*-diphenols (r = 0.69), flavonoids (r = 0.77,), ABTS•^+^ (r = 0.53), DPPH (r = 0.54), and FRAP (r = 0.75). Moreover, the same was observed for both *ortho*-diphenols and flavonoids, who also presented statistically significant correlations (*p* < 0.05) with all variables. Specifically, *ortho*-diphenols correlated strongly with ABTS•^+^ (r = 0.67, *p* < 0.05) and DPPH (r = 0.71, *p* < 0.05), while flavonoids also displayed strong associations with ABTS•^+^ (r = 0.67, *p* < 0.05) and DPPH (r = 0.69, *p* < 0.05). Within individual phenolics, gallic acid correlated positively with all colorimetric assays, with strong correlations being observed with TPC (r = 0.55, *p* < 0.05) and FRAP (r = 0.65, *p* < 0.05). It also exhibited strong correlation to other individual compounds, especially caffeic acid (r = 0.73, *p* < 0.05) and epicatechin (r = 0.74, *p* < 0.05), with all three increasing simultaneously in most varieties under study in 2022. Remarkably, this was also observed for caftaric acid with catechin, which were highly correlated (r = 0.96, *p* < 0.05), and increased in conjunction in this work. Among flavonols, namely myricetin, quercetin, and isorhamnetin derivatives, these generally revealed statistically significant (*p* < 0.05) moderate correlations with TPC and the antioxidant activity assays (ranging from r = 0.23 to 0.54). Nonetheless, they correlated notably with each other, for example, myricetin-3-*O*-glucoside and quercetin-3-*O*-galactoside (r = 0.45, *p* < 0.05) and quercetin-3-*O*-galactoside and quercetin-3-*O*-glucoronide (r = 0.49, *p* < 0.05). Moreover, isorhamnetin-3-*O*-glucoside presented positive correlations with many of these same flavonols and TPC, although its association with ABTS•^+^ and catechin were the only ones being not significant in this analysis. In fact, the positive correlations between flavonols indicates that they increase together.

### 2.10. Principal Component Analysis

**Figure 9:**
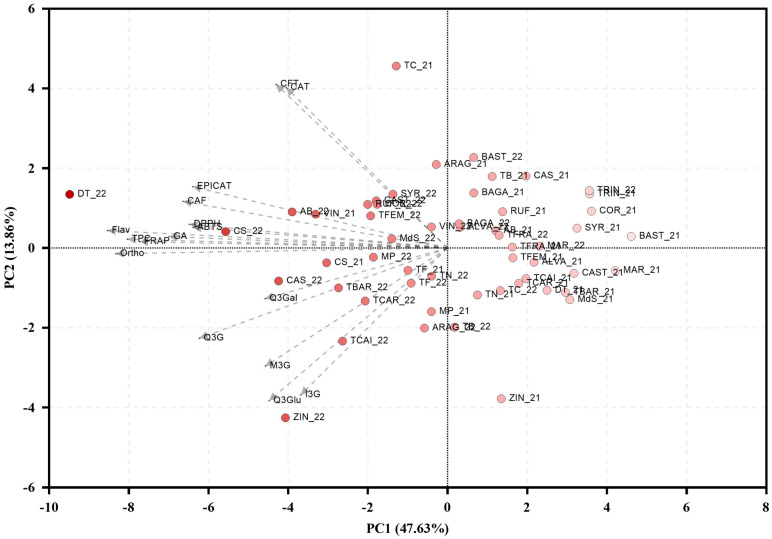
Principal component analysis (PCA) integrating all phenolic and antioxidant parameters quantified in the 27 red grapevine varieties under study in 2021 and 2022. PC—Principal component; Years: _21—2021; _22—2022; Varieties: AB—Alicante Bouschet; ARAG—Aragonez; ALVA—Alvarelhão; BAGA—Baga; BAST—Bastardo; CAS—Casculho; CAST—Castelão; COR—Cornifesto; CS—Cabernet Sauvignon; DT—Donzelinho Tinto; MAR—Marufo; MdS—Mourisco de Semente; MP—Malvasia Preta; RUF—Rufete; SYR—Syrah; TB—Tinta Barroca; TBAR—Tinta da Barca; TC—Tinto Cão; TCAI—Tinta Caiada; TCAR—Tinta Carvalha; TF—Touriga Franca; TFEM—Touriga Fêmea; TFRA—Tinta Francisca; TN—Touriga Nacional; TRIN—Trincadeira; VIN—Vinhão; ZIN—Zinfandel. Parameters: CAT—catechin; CAF—Caffeic acid; CFT—Caftaric acid; ECAT—Epicatechin; Flav—Flavonoids; GA—Gallic acid; I3G—isorhamnetin-3-*O*-glucoside; M3G—malvidin-3-*O*-glucoside; Ortho—*ortho*-diphenols; Q3Gal—quercetin-3-*O*-galactoside; Q3G—quercetin-3-*O*-glucoside; Q3Glu—Quercetin-3-*O*-glucuronide.

A principal component analysis (PCA) integrating all data of the evaluated parameters was performed (Figure 9). In this analysis, the first two principal components (PC1 and PC2) together explained 61.50% of the total variance, with PC1 accounting for 47.63% and PC2 13.86%. Parameters such as TPC, *ortho*-diphenols, flavonoids, and FRAP exhibited a strong alignment along PC1, grouping closely with gallic acid, caffeic acid, and epicatechin, and forming a dense cluster in the left quadrant of the PCA plot. Conversely, variables such as catechin, and quercetin-3-*O*-glucuronide were more closely associated with PC2. Although no distinct clusters were observed, most of the 2022 samples were distributed along the left side of the PCA plot, in the upper left and lower left quadrants, whereas the 2021 samples predominantly aligned with the upper and lower right quadrants.

## 3. Discussion

The phenolic composition of grapes is of utmost importance when it comes to wine quality [20,32], with their biosynthesis being dependent on the adaptative response of the plant [33]. Despite phenolic composition in red grapes being predominantly dominated by their anthocyanin fraction [34,35], other phenolic compounds significantly contribute to both wine quality and stress resilience. Understanding how their concentration varies among varieties as well as growing seasons is essential for optimizing grape cultivation and wine production. To address this, the phenolic profile of 27 red grape varieties was analyzed over two consecutive growing seasons in Portugal’s Douro Demarcated Region. The study aimed to assess how the total phenolic content, specific phenolic subclasses, and antioxidant activity of different grapevine varieties change with climatic variations.

Total phenolic content (TPC) was observed to vary significantly between varieties as well as between years (*p* < 0.001, Figure 2), with ‘Vinhão’ and ‘Donzelinho Tinto’ exhibiting the highest contents in 2021 and 2022 respectively. Differences in the quantification of phenolic compounds among red grape varieties are a well-documented occurrence, which can usually be attributed to genetic differences [36,37,38,39,40]. Among all varieties, ‘Aragonez’, ‘Marufo’, ‘Tinta Barroca’, ‘Tinta Francisca’, ‘Tinto Cão’, ‘Touriga Franca’, ‘Trincadeira’, and ‘Vinhão’ remained statistically unchanged between years. Nonetheless, this general increase in TPC between years could be attributed to the contrasting climatic conditions of both years, as 2022 was warmer and dryer than 2021 (Figure 2). In fact, the extended abiotic stress experienced could have led to increases in phenolic synthesis and accumulation [30,41,42], as these compounds act as primary antioxidants, scavenging ROS generated and accumulated under heat and drought stress to mitigate oxidative damage [24,25,43]. Notably, ‘Donzelinho Tinto’ displayed an exceptional plasticity, doubling its TPC in 2022, while others, such as ‘Bastardo’ and ‘Tinta Barroca’, showed minimal TPC changes. These differing behaviors further highlight how, despite the general increase in TPC observed, there are still differences among germplasm that could aid in the selection of well-adapted varieties [14,15]. Similar to TPC, *ortho*-diphenol content varied significantly across the 27 red grape varieties and between growing seasons. The highest levels were observed in ‘Vinhão’ in 2021 and ‘Donzelinho Tinto’ in 2022, while ‘Trincadeira’ exhibited the lowest values in both years. The substantial increase in *ortho*-diphenol content in 2022 aligns with previous research indicating that these compounds can accumulate in response to abiotic stress, serving as strong protective agents against oxidative damage [44,45]. In fact, the statistically significant (*p* < 0.05) strong positive correlation between *ortho*-diphenol content and antioxidant activity across the three assays (ABTS•^+^, DPPH, and FRAP) observed in this work further reinforces their role in ROS scavenging and oxidative stress mitigation [46]. Nonetheless, while most varieties showed an increase in *ortho*-diphenols in 2022, ‘Tinto Cão’ and ‘Vinhão’ experienced a statistically significant decline. This suggests that, while most grape varieties are capable of increasing their phenolic content under stress conditions, certain cultivars may exhibit a threshold beyond which phenolic biosynthesis becomes constrained, or resources are redirected to other branches or pathways of stress-response metabolism, such as lignin or stilbene synthesis, osmoprotectant production, or hormone-mediated signaling [23,47,48]. Regarding flavonoid content, it increased across most varieties (Figure 4). Notably, ‘Cabernet Sauvignon’ displayed the highest flavonoid levels, while ‘Donzelinho Tinto’ exhibited the highest concentration in 2022. Flavonoid biosynthesis is largely governed by genetic factors, while also being highly responsive to environmental stressors such as heat and drought [17,47]. In fact, elevated temperatures have been linked to increased flavonol and stilbene accumulation, with these compounds playing crucial roles in photoprotection and antioxidant defense mechanisms [49]. Thus, the increases observed between both years likely indicate both a common genetic predisposition and a direct photoprotective response to the more stressful conditions. Nonetheless, ‘Tinto Cão’ exhibited a notable decline, mirroring its response in *ortho*-diphenol content, suggesting differences in stress response. Moreover, ‘Aragonez’, ‘Baga’, ‘Cabernet Sauvignon’, ‘Malvasia Preta’, ‘Tinta Carvalha’, ‘Tinta Francisca’, ‘Touriga Fêmea’, ‘Touriga Franca’, ‘Touriga Nacional’, and ‘Trincadeira’ also did not reveal any significant changes. As flavonoids can be indicators of adaptability to climate stress, varieties that maintain or increase their flavonoid content under stress conditions may be less vulnerable to oxidative damage [46,47,49].

HPLC-DAD analysis of individual phenolic compounds (Table 1 and Table 2) allowed us to identify 10 major compounds, with quercetin derivates being the most common. Gallic acid was the only hydroxycinnamic acid identified, revealing a significant accumulation in 2022 amongst most varieties, particularly in ‘Donzelinho Tinto’. Increases in this acid have been observed in grape varieties exposed to water stress [50], with it contributing to oxidative stress defense, ameliorating the effects of abiotic stress [51]. In fact, stress-induced shifts in gallic acid are commonly documented in plant species with differing drought and heat tolerances, where tolerant genotypes often accumulate higher levels than sensitive ones, suggesting a conserved protective role across taxa [52,53]. Thus, the accumulation of this compound in most varieties could suggest a common adaptive metabolic response aimed at mitigating oxidative damage caused by the increased heat and drought experienced in 2022. Notably, as gallic acid is a potent antioxidant, the overall increase in antioxidant activity detected by the FRAP assay may be partly associated with its increased levels, which is supported by their strong positive correlation (r = 0.65, Figure 8). Nonetheless, this trend was not universal, as ‘Trincadeira’ and ‘Tinto Cão’ exhibited minimal or negative changes in gallic acid concentration. Hydroxycinnamic acids are also known to be integral in grapevine protection against abiotic stress, despite their accumulation behavior not being fully understood [54]. In this study we quantified both caftaric acid and caffeic acid and observed their variation to be highly varietal-dependent (Table 1). For instance, ‘Tinto Cão’ experienced a 66% decline in caftaric acid levels, while ‘Bastardo’ and ‘Castelão’ exhibited significant increases. These differences could suggest a differing upregulation of key enzymes in the phenylpropanoid pathway such as phenylalanine ammonia-lyase, which could indicate a more efficient protective mechanism against environmental stress [46]. Similarly to caftaric acid, caffeic acid concentration also increased across most varieties. The common decreases in phenolic acids observed in the ‘Tinto Cão’ variety could also explain the general decrease in the antioxidant activity observed for this variety, as this group of compounds is tightly connected to antioxidant potential [19,53,55].

Flavan-3-ols, namely catechin and epicatechin play a dual function by influencing both astringency in wine and oxidative stress mitigation mechanisms in the grape berry [20,56,57]. In this study, their concentrations varied markedly among varieties and across years, underscoring contrasting metabolic trade-offs (Table 1). For instance, ‘Tinto Cão’ experienced severe declines in catechin and epicatechin from 2021 to 2022, while ‘Donzelinho Tinto’ exhibited remarkable increases, especially in epicatechin. These results hint at different genetic predisposition between varieties under the same growing conditions. This has also been previously observed by Pinasseau et al. [58], who observed that different grapevine varieties under drought stress can accumulate and present different patterns of polyphenols. These dynamics also have practical implications for winemaking and aging, since varieties high in monomeric flavan-3-ols may afford greater antioxidant resilience but exhibit milder astringency [20,39]. Flavonols are highly recognized for their role in photoprotection by absorbing harmful UV radiation, while also mitigating oxidative stress cause by high temperatures and drought in grape tissues [55,59,60,61]. In this study, we observed that myricetin and quercetin derivatives were the most common flavonols, which aligns with previous studies [31,62]. Specifically, quercetin derivatives showed relative increased concentrations under the 2022 conditions, despite ‘Tinto Cão’, ‘Tinta Barroca’, and ‘Casculho’ diverging from this pattern. These differences highlight how grapevine varieties differ amongst them, suggesting they might differ in the regulation of enzymatic routes involved in flavonol biosynthesis [63]. Beyond these varietal differences, it is also important to recognize that phenolic accumulation is further shaped by broader terroir-related influences and their interactions, including other environmental conditions as well as vineyard management practices. While this present work highlights clear genetic and interannual effects, phenolic biosynthesis is highly plastic and can be further modulated by vineyard-specific conditions such as soil properties, water availability, canopy architecture, berry exposure, and crop load [14,23,64]. Consequently, the patterns observed in this study may not remain entirely stable across different sites or under alternative viticultural practices, particularly when environmental constraints differ markedly, something that has been documented in both Mediterranean and non-Mediterranean regions [39,65,66,67]

Antioxidant activity was assessed via ABTS•^+^, DPPH, and FRAP assays (Figure 5, Figure 6 and Figure 7). Similarly to the quantification of phenolic compounds, ABTS•^+^ and DPPH radical scavenging activities were influenced by both genetic factors and environmental conditions, with interannual variations largely reflecting the impact of abiotic stressors. On the other hand, the FRAP assay revealed a rather dominant year effect, more-so than varietal. Contrarily to what was observed in the phenolic quantification, antioxidant activity did not follow an obvious pattern. For example, ‘Casculho’, ‘Castelão’, ‘Donzelinho Tinto’, ‘Mourisco de Semente’, ‘Syrah’, ‘Tinta Carvalha’, ‘Tinta da Barca’, ‘Touriga Nacional’, and ‘Zinfandel’ increased significantly in ABTS•^+^ antioxidant activity, whereas ‘Aragonez’, ‘Baga’, ‘Cabernet Sauvignon’, ‘Tinta Francisca’, ‘Tinto Cão’, and ‘Vinhão’ dropped. Despite these variances, all assays exhibited high positive correlations with the quantified phenolic compounds (Figure 8). Differences in antioxidant activity between varieties, along with differences in phenolic composition, are not uncommon and have previously been observed in similar works [38,39,68,69]. For instance, while ‘Cabernet Sauvignon’, and ‘Vinhão’ exhibited somewhat consistent high antioxidant potential across the three assays, despite the differences between years, varieties such as ‘Bastardo’, ‘Marufo’ and ‘Trincadeira’, showed relatively low antioxidant activity, which could suggest a more limited capacity to upregulate phenolic-based defenses [17,46]. This could be the reason why some varieties exhibited dramatic changes, including ‘Donzelinho Tinto’ and ‘Zinfandel’, which also aligned with their increased accumulation of total phenolics, flavonoids, and *ortho*-diphenols under the warmer and drier conditions of 2022. Conversely, ‘Tinto Cão’ and ‘Vinhão’ experienced significant declines in antioxidant activity despite increased environmental stress, indicating potential metabolic limitations or shifts in resource allocation within these cultivars. The interannual shifts, mainly the increase in phenolic concentration and antioxidant activity observed in most varieties are consistent with the established roles of phenolics in grapevine stress response. These compounds contribute directly to ROS scavenging, limiting oxidative damage generated during heat and drought stress [64]. For instance, flavonols can help reduce photoinhibition and protect cellular structures under high abiotic stress conditions, with recent studies showing their regulation interplays with antioxidant enzymes [70]. Additionally, several phenolic subclasses, such as hydroxybenzoic and hydroxycinnamic acids, support drought-tolerance mechanisms by stabilizing cell membranes, modulating stomatal behavior, and contributing to osmotic adjustment [53,71]. Thus, the common increase observed in 2022 likely reflected the increased activation of the phenylpropanoid pathway as grapevines responded to elevated temperatures and reduced water availability.

The Pearson correlation analysis (Figure 7) revealed strong positive correlations between total phenolic content and each antioxidant parameter, confirming that higher levels of phenolics generally confer superior radical-scavenging and reducing capacities [29,30,69]. Furthermore, certain phenolics, such as gallic acid, caffeic acid, and epicatechin, showed particularly high mutual correlations, suggesting these compounds may be regulated by overlapping biosynthetic pathways or respond similarly to the same environmental conditions. The strong associations between phenolic compounds and antioxidant activity underscore the importance of phenolic composition in maintaining grape berry redox balance under stress. Finally, PCA analysis (Figure 9) supported the correlation results by highlighting consistent associations. Although no clear varietal clusters emerged, most 2022 samples grouped separately from 2021, being mostly distributed along the left quadrants, indicating that warmer, drier conditions led to a coordinated shift in phenolic profiles and antioxidant traits across varieties.

## 4. Materials and Methods

### 4.1. Plant Material and Growth Conditions

For this work, 27 different red grapevine (*V. vinifera* L.) varieties, namely: ‘Alicante Bouschet’, ‘Alvarelhão’, ‘Aragonez’, ‘Baga’, ‘Bastardo’, ‘Cabernet Sauvignon’, ‘Casculho’, ‘Castelão’, ‘Cornifesto’, ‘Donzelinho Tinto’, ‘Malvasia Preta’, ‘Marufo’, ‘Mourisco-de-Semente’, ‘Rufete’, ‘Syrah’, ‘Tinta Caiada’, ‘Tinta Carvalha’, ‘Tinta da Barca’, ‘Tinta Francisca’, ‘Tinta-Barroca’, ‘Tinto Cão’, ‘Touriga Fêmea’, ‘Touriga Franca, ‘Touriga Nacional’, ‘Trincadeira’, ‘Vinhão’, and ‘Zinfandel’ were sampled from a grapevine variety library located in the North of Portugal, Vale da Vilariça, Bragança, at Quinta do Ataíde, Symington Family Estates (41°14′ N, 7°6′ W, 125 m). All plants were established in 2014 with a spacing of 2.2 m × 1 m in a Royat single cordon training system with vertical shoot positioning, grown under the same conditions, and subjected to the same cultural practices. Sampling occurred during the growing seasons of 2021 and 2022 at the harvest time, established by the technological maturity criteria defined by the producer, by randomly collecting 5 fresh berries from 4 different bunches from 3 selected grapevines of each variety (5 berries × 4 bunches × 3 vines × 27 varieties), flash freezing them and immediately storing at −80 °C until the lyophilization process.

### 4.2. Quantification of Different Phenolic Compounds

#### 4.2.1. Extraction of Phenolic Compounds

Hydro-methanolic extracts were obtained as described by Gouvinhas et al. [72] and Mendes Lemos et al. [73]. In sum, 1.5 mL of a 70:30 (*v*/*v*) methanol/ultra-pure water solution was added to 40 mg of lyophilized material, vortexed and agitated at room temperature for 30 min. Following this, this mixture was centrifuged at 10,000× *g* rpm for 15 min at 4 °C and the supernatant collected. This procedure was repeated until a final concentration of 4 mg·mL^−1^. Extracts were stored at −20 °C and used for the determination of the total phenolics, flavonoids, ortho-diphenols and antioxidant activity assays.

#### 4.2.2. Determination of Total Phenolic Content

Total phenolic content (TPC) was determined using the Folin-Ciocalteu colorimetric method described by Singleton and Rossi [74] with slight modifications. In sum, 20 µL of the previously obtained extract were combined with 100 µL of Folin-Ciocalteu reagent (diluted 1:10) and 80 µL of 7.5% sodium carbonate solution in a 96-well microplate. Samples were left to incubate in the dark for 30 min, followed by the absorbance reading at 765 nm. A calibration curve with gallic acid concentrations was used to quantify the phenolic content. Results were expressed as mg of gallic acid equivalents per gram of dry weight (mg GAE·g^−1^ DW).

#### 4.2.3. Determination of Ortho-Diphenol Content

*Ortho*-diphenols content was determined following the protocol described by Gouvinhas et al. [72]. In sum, 160 µL of extract were added 40 µL of Na_2_MoO_4_ solution and incubated in the dark for 15 min in a 96-well microplate, before measuring absorbance at 370 nm. *Ortho*-diphenolic content was expressed as mg of gallic acid equivalents per g of dry weight (mg GAE·g^−1^ DW) using a gallic acid calibration curve.

#### 4.2.4. Determination of Flavonoid Content

Flavonoid content was evaluated following the methodology described by Dewanto et al. [75] with slight modifications. For this, 25 µL of extract were mixed with 100 µL of ultra-pure water and 10 µL of 5% NaNO_2_ and incubated at room temperature in the dark for 5 min. After, 15 µL of 10% AlCl_3_ were carefully added and the microplate incubated for 6 min. Finally, 50 µL of 1 M NaOH and 50 µL of ultra-pure water were added and absorbance measured at 510 nm. Results were expressed as mg of catechin equivalents per gram of dry weight (mg CE·g^−1^ DW) using a standard curve of catechin at various concentrations.

### 4.3. Analysis of Antioxidant Activity

#### 4.3.1. ABTS•^+^ Radical-Scavenging Activity

The ABTS•^+^ (2,2′-azino-bis (3-ethylbenzothiazoline-6-sulphonic acid)) radical-scavenging activity was measured following the methodology described by Re et al. [76] and Stratil et al. [77]. In sum, a working solution of ABTS•^+^ was prepared by mixing 7 mM ABTS•^+^ with 140 mM K_2_S_2_O_8_ in ultra-pure water and leaving the solution in the dark for 12–16 h at room temperature. Absorbance of the working solution was adjusted to be around 0.7–0.8 at 734 nm. For sample analysis, 188 µL of ABTS•^+^ solution were added 12 µL of extract, incubated in the dark for 10 min, and absorbance measured at 734 nm. A Trolox calibration curve was prepared, and results expressed as mg of Trolox equivalents per g of dry weight (mg TE·g^−1^ DW).

#### 4.3.2. DPPH Radical-Scavenging Activity

For DPPH (2,2-Diphenyl-1-picrylhydrazyl) radical-scavenging activity, the methodology used was as described by Siddhuraju and Becker [78]. In sum, 10 µL of extract were added 190 µL of a methanolic DPPH (60 µM) solution and vigorously shaken. After incubation in the dark 30 min, absorbance was measured at 517 nm, and results expressed as mg of Trolox equivalents per g of dry weight (mg TE·g^−1^ DW) using a Trolox calibration curve.

#### 4.3.3. FRAP Assay

For the Ferric Reducing Antioxidant Power (FRAP) assay, the methodology used was adapted from Stratil et al. [77] and Mena et al. [79]. For this, FRAP reagent was prepared by mixing 1 volume of 10 mM TPTZ (2,4,6-tripyridyl-s-triazine) in 40 mM HCl with 1 volume of 20 mM FeCl_3_·6H_2_O and 10 volumes of 300 mM acetate buffer at pH 3.6. After, 25 µL of extract were added 275 µL of FRAP reagent, vigorously shaken and incubated in the dark for 5 min. Absorbance was measured at 593 nm, and results expressed as mg of Trolox equivalents per g of dry weight (mg TE·g^−1^ DW) using a Trolox calibration curve.

### 4.4. Non-Anthocyanin Phenolic Profile by HPLC-DAD

#### 4.4.1. Extraction Procedure

Extraction for the HPLC-DAD analysis of non-anthocyanin profiles was performed using 200 mg of fine grape berry powder and adding 1 mL of methanol:water (70:30, *v*/*v*). Each mixture was set in an ultrasonic bath for 30 min and centrifuged at 8000× *g* rpm for 15 min at 4 °C. The supernatant was collected and filtered to a vial using a 0.22 µm Nylon membrane filter and stored at −20 °C until the analysis.

#### 4.4.2. Chromatography Analysis Conditions

High-performance liquid chromatography with diode array detection (HPLC-DAD) analyses were performed using a Thermo Scientific Dionex Ultimate 3000 UHPLC system (Thermo Fisher Scientific, Bremen, Germany) equipped with a quaternary gradient pump, an autosampler, a column oven, and a diode array detector. Autosampler sample tray was kept at 25 °Cand separation of the compounds performed in a ProntoSIL 120-5-C18 ace-EPS column (250 mm × 4.6 mm, 5 μm; BISCHOFF Chromatography, Leonberg, Germany). at 35 °C. Mobile phases consisted of water/trifluoracetic acid (99.9:0.1, *v*/*v*, phase A) and acetonitrile/trifluoracetic acid (99.9%:0.01, *v*/*v*, phase B). Flow rate was 0.8 mL·min^−1^, and solvent gradient was as follows (t min:% B): 0 min, 10% B; 5 min:20% B; 25 min:35% B; 30 min:50% B; 35 min:100% B; 45 min:10% B; for a total run time of 45 min. Column was equilibrated for 5 min between injections. The injection volume was 10 μL, and identification and quantification of non-anthocyanin phenolic compounds was made with DAD-chromatograms at 280 nm, 320 nm and 370 nm. Phenolic compounds were identified according to their retention times and UV-Vis against those of standard compounds. Calibration curves of each respective standard were used for the quantification of each compound. When the respective standard was not available, the calibration curve of the closest similar compound was used.

### 4.5. Statistical Analysis

Statistical analyses were conducted using SPSS Statistics for Windows, version 23.0 (IBM Corp., Armonk, NY, USA). The assumptions of normality and homogeneity of variances were verified using the Shapiro–Wilk and Levene’s tests, respectively. A two-way ANOVA was conducted to evaluate the effects of year and variety, followed by Tukey’s post hoc test to identify significant differences (*p* < 0.05), with significant results being represented as follows: *—*p* < 0.05; **—*p* < 0.01; ***—*p* < 0.001.

To assess variable relationships and reduce dimensionality, Pearson correlation and principal component analysis (PCA) were performed using GraphPad Prism version 10.4.0 (GraphPad Software, San Diego, CA, USA). For the PCA, components with eigenvalues above 1 were retained, and the contribution of each variable was evaluated based on factor loadings.

## 5. Conclusions

This study demonstrated significant varietal differences in the non-anthocyanin phenolic composition and antioxidant capacity of 27 red grapevine varieties cultivated in northern Portugal. The results indicate that environmental factors, particularly temperature and precipitation can strongly influence phenolic accumulation. The warmer and drier 2022 season led to increased total phenolic content and antioxidant activity in most varieties. These findings reinforce the central role of phenolics in grapevine stress physiology and highlight significant varietal differences in phenolic plasticity. Varieties such as ‘Donzelinho Tinto’, ‘Vinhão’, ‘Zinfandel’, and ‘Casculho’ exhibited pronounced interannual increases, indicating a strong capacity to upregulate phenolic-based defenses when exposed to abiotic stress. Conversely, varieties such as ‘Tinta Francisca’, ‘Touriga Franca’, and ‘Trincadeira’ remained comparatively stable across both growing seasons, suggesting a degree of phenolic homeostasis. These contrasting strategies highlight the importance of both plastic and stable varieties in viticultural planning, depending on whether adaptive responsiveness or compositional consistency is desired. The strong correlations observed between individual phenolic groups and antioxidant assays further demonstrate how phenolic composition shapes the oxidative resilience of grape berries, a trait that is relevant not only for vine performance under climate stress but also for wine quality and aging potential. Collectively, these results emphasize the value of autochthonous germplasm as a reservoir of climate-adaptive traits and support its integration into sustainable vineyard management and varietal selection strategies. Nonetheless, future research should deepen our knowledge of genotype–environment interactions across multiple sites and seasons, integrating additional physiological and agronomic indicators, to refine the identification of varieties best suited to emerging climatic scenarios and to support more resilient viticultural systems.

## Figures and Tables

**Table 1:** Hydroxybenzoic acids, hydroxycinnamic acids and flavan-3-ols in the berry extracts of the varieties under study. Results are expressed as µg·g^−1^ DW. Values presented as mean ± standard deviation. For statistically significant differences between years within varieties: *—*p* < 0.05; **—*p* < 0.01; ***—*p* < 0.001. *p*(V), *p*(Y), and *p*(V × Y) correspond to the *p* values of the main effects of variety, year, and their interaction. V—variety; Y—year; LOQ—below the limit of quantification.

Variety	Year	Hydroxybenzoic Acids	Hydroxycinnamic Acids		Flavan-3-ols	
		Gallic Acid	Caftaric Acid	Caffeic Acid	Catechin	Epicatechin
‘Alicante Bouschet’	2021	24.69 ± 5.51 ***	14.11 ± 6.64 **	13.26 ± 1.94 ***	66.12 ± 29.94	69.68 ± 39.17 ***
	2022	48.20 ± 18.57 ***	27.38 ± 4.67 **	73.85 ± 16.57 ***	127.75 ± 28.83	414.52 ± 102.23 ***
‘Alvarelhão’	2021	13.01 ± 6.14	14.74 ± 1.69	20.22 ± 3.50	49.74 ± 10.45	37.99 ± 13.79
	2022	16.17 ± 5.83	20.19 ± 1.38	23.75 ± 6.87	83.38 ± 8.53	105.32 ± 42.42
‘Aragonez’	2021	6.88 ± 5.90	33.47 ± 9.02 **	22.18 ± 2.45	165.33 ± 55.68 **	45.86 ± 12.38
	2022	12.38 ± 4.94	18.02 ± 6.47 **	21.32 ± 2.30	69.96 ± 39.94 **	41.75 ± 7.32
‘Baga’	2021	10.55 ± 3.92	25.24 ± 8.53	13.16 ± 1.94	114.50 ± 52.65	64.92 ± 7.02
	2022	15.91 ± 3.17	19.88 ± 3.87	23.32 ± 14.34	81.42 ± 23.87	69.34 ± 34.28
‘Bastardo’	2021	9.19 ± 3.37 *	9.02 ± 0.50 *	8.83 ± 0.16 ***	14.43 ± 3.09 *	84.63 ± 19.47 ***
	2022	23.95 ± 5.08 *	20.97 ± 9.63 *	55.53 ± 44.77 ***	88.14 ± 59.42 *	368.11 ± 184.82 ***
‘Cabernet Sauvignon’	2021	21.75 ± 14.20	17.40 ± 6.89	11.53 ± 0.22 ***	53.36 ± 21.17 ***	404.29 ± 147.61
	2022	29.30 ± 6.46	26.78 ± 3.54	81.98 ± 32.60 ***	165.2 ± 90.35 ***	464.70 ± 201.17
‘Casculho’	2021	6.42 ± 4.07 ***	30.47 ± 11.61 *	21.87 ± 8.00 *	146.78 ± 71.62 *	94.12 ± 61.09 *
	2022	26.72 ± 4.67 ***	19.91 ± 4.45 *	45.85 ± 19.71 *	81.64 ± 27.45 *	241.71 ± 121.61 *
‘Castelão’	2021	5.95 ± 3.86	11.20 ± 1.60 *	12.35 ± 1.09 *	27.90 ± 9.86 *	59.09 ± 26.46 *
	2022	17.60 ± 2.98	21.98 ± 7.67 *	36.72 ± 13.55 *	94.38 ± 47.35 *	185.40 ± 83.61 *
‘Cornifesto’	2021	4.55 ± 3.07 **	14.56 ± 2.77	20.84 ± 6.11	48.62 ± 17.11	52.50 ± 25.24
	2022	22.61 ± 5.79 **	22.99 ± 5.96	28.81 ± 13.52	100.62 ± 36.77	136.58 ± 83.43
‘Donzelinho Tinto’	2021	12.18 ± 3.07 ***	12.00 ± 2.52 ***	12.09 ± 2.84 ***	32.82 ± 15.53 *	65.96 ± 15.18 ***
	2022	69.96 ± 9.78 ***	30.20 ± 11.47 ***	157.06 ± 36.56 ***	111.81 ± 34.82 *	928.04 ± 225.60 ***
‘Malvasia Preta’	2021	7.98 ± 7.65 **	14.24 ± 3.04	16.99 ± 1.38	46.66 ± 18.78	63.91 ± 24.17
	2022	24.70 ± 8.83 **	17.10 ± 4.51	25.24 ± 4.83	64.27 ± 27.82	114.55 ± 29.79
‘Marufo’	2021	8.51 ± 3.85 **	12.18 ± 0.90	14.78 ± 3.22	33.92 ± 5.58	37.31 ± 11.13
	2022	24.88 ± 9.71 **	17.30 ± 8.47	24.43 ± 16.09	65.53 ± 52.29	109.51 ± 99.31
‘Mourisco-de-Semente’	2021	3.82 ± 1.79 **	10.53 ± 1.84 *	15.44 ± 6.58	35.52 ± 5.25 *	69.79 ± 29.14
	2022	21.55 ± 4.77 **	24.5 ± 5.02 *	20.13 ± 1.16	109.95 ± 30.96 *	83.00 ± 7.13
‘Rufete’	2021	26.68 ± 12.95	12.45 ± 1.35	16.19 ± 2.60 ***	35.58 ± 8.32	266.32 ± 132.83
	2022	36.10 ± 8.33	17.34 ± 2.13	57.30 ± 10.72 ***	65.79 ± 13.17	312.36 ± 66.18
‘Syrah’	2021	5.74 ± 4.73 ***	16.24 ± 2.12	14.27 ± 0.90 ***	59.01 ± 13.09	126.19 ± 118.35 *
	2022	32.82 ± 16.16 ***	20.44 ± 10.34	47.25 ± 15.90 ***	84.91 ± 63.79	250.33 ± 98.11 *
‘Tinta Barroca’	2021	6.60 ± 3.51	32.66 ± 11.31 ***	13.92 ± 1.83	160.33 ± 69.81 ***	40.34 ± 10.17
	2022	7.05 ± 4.01	14.42 ± 5.57 ***	19.24 ± 0.63	47.75 ± 34.37 ***	107.58 ± 18.32
‘Tinta Caiada’	2021	9.90 ± 1.71 *	11.72 ± 2.69	11.85 ± 2.00	31.07 ± 16.57	15.10 ± 5.76
	2022	25.51 ± 2.74 *	15.38 ± 0.96	13.26 ± 0.64	53.66 ± 5.95	40.61 ± 3.98
‘Tinta Carvalha’	2021	15.44 ± 4.09 *	13.81 ± 2.10	14.42 ± 1.72 *	43.97 ± 12.96	142.29 ± 77.23
	2022	30.21 ± 10.65 *	19.70 ± 6.87	35.53 ± 7.03 *	80.31 ± 42.41	178.03 ± 43.40
‘Tinta da Barca’	2021	4.75 ± 5.21 ***	12.61 ± 0.51	14.41 ± 0.48	36.57 ± 3.17	35.69 ± 19.55 *
	2022	28.39 ± 10.06 ***	15.24 ± 1.47	35.11 ± 7.04	52.81 ± 9.07	175.42 ± 43.43 *
‘Tinta Francisca’	2021	4.62 ± 3.09	11.68 ± 4.45	16.90 ± 1.83	30.82 ± 27.46	74.25 ± 26.71
	2022	5.43 ± 0.59	21.20 ± 10.14	11.11 ± 2.13	89.57 ± 62.58	19.98 ± 15.71
‘Tinto Cão’	2021	11.59 ± 9.38	43.70 ± 9.85 ***	17.71 ± 5.24	228.47 ± 60.77 ***	232.53 ± 179.88 *
	2022	5.40 ± 0.42	14.84 ± 3.61 ***	17.33 ± 1.16	50.36 ± 22.28 ***	96.15 ± 61.00 *
‘Touriga Fêmea’	2021	5.79 ± 6.00 ***	12.09 ± 3.44 *	22.35 ± 3.12	33.39 ± 21.20 **	90.42 ± 11.67
	2022	29.46 ± 5.58 ***	23.63 ± 8.81 *	27.58 ± 8.67	132.97 ± 68.76 **	118.50 ± 27.28
‘Touriga Franca’	2021	4.96 ± 4.01	22.80 ± 10.20	15.77 ± 1.49	99.44 ± 62.92	56.34 ± 27.80
	2022	5.32 ± 1.04	20.10 ± 7.64	17.45 ± 2.99	82.78 ± 47.13	47.41 ± 9.94
‘Touriga Nacional’	2021	14.46 ± 16.41	13.08 ± 2.78	19.94 ± 0.47	49.35 ± 1.27	58.42 ± 24.05
	2022	7.00 ± 3.88	14.64 ± 1.12	16.72 ± 1.37	49.10 ± 6.89	35.61 ± 26.22
‘Trincadeira’	2021	13.01 ± 6.31	21.35 ± 2.78	9.32 ± 1.55	90.50 ± 17.16	33.31 ± 20.37
	2022	4.41 ± 1.78	21.82 ± 8.28	19.46 ± 12.98	93.39 ± 51.11	82.01 ± 47.36
‘Vinhão’	2021	14.06 ± 3.90	30.02 ± 1.77	18.5 ± 1.14	144.03 ± 10.89	57.54 ± 24.32
	2022	12.50 ± 7.23	22.24 ± 8.08	16.38 ± 4.36	96.00 ± 49.84	59.86 ± 26.88
‘Zinfandel’	2021	9.52 ± 6.36 ***	12.32 ± 2.53	12.16 ± 0.78 **	34.80 ± 15.62	42.11 ± 5.88 **
	2022	42.70 ± 9.24 ***	17.04 ± 4.05	40.02 ± 6.02 **	63.95 ± 24.96	205.76 ± 37.16 **
*p*(V)		<0.001	<0.001	<0.001	<0.001	<0.001
*p*(Y)		<0.001	<0.05	<0.001	<0.05	<0.001
*p*(V × Y)		<0.001	<0.001	<0.001	<0.01	<0.001

**Table 2:** Flavonols in the berry extracts of the varieties under study. Results are expressed as µg·g^−1^ DW. Values presented as mean ± standard deviation. For statistically significant differences between years within varieties: *—*p* < 0.05; **—*p* < 0.01; ***—*p* < 0.001. *p*(V), *p*(Y), and *p*(V × Y) correspond to the *p* values of the main effects of variety, year, and their interaction. V—variety; Y—year; LOQ—below the limit of quantification.

Variety	Year	Flavonols				
		Myricetin-3-*O*-Glucoside	Quercetin-3-*O*-Galactoside	Quercetin-3-*O*-Glucoronide	Quercetin-3-*O*-Glucoside	Isorhamnetin-3-*O*-Glucoside
‘Alicante Bouschet’	2021	164.37 ± 71.95 *	14.39 ± 4.30	164.18 ± 89.73 *	97.22 ± 36.23	61.62 ± 19.19
	2022	300.80 ± 48.44 *	32.60 ± 6.46	388.00 ± 62.36 *	204.61 ± 82.64	102.24 ± 12.83
‘Alvarelhão’	2021	55.23 ± 9.02	32.80 ± 14.42	394.40 ± 178.49	172.19 ± 8.76	42.33 ± 9.89
	2022	103.74 ± 61.15	25.16 ± 2.71	311.27 ± 30.04	296.89 ± 102.95	29.58 ± 4.66
‘Aragonez’	2021	241.15 ± 64.43	24.66 ± 8.65	234.14 ± 100.47 *	179.82 ± 36.94	48.44 ± 15.05 **
	2022	360.80 ± 75.93	36.70 ± 3.97	503.92 ± 185.16 *	292.87 ± 21.31	112.91 ± 49.28 **
‘Baga’	2021	230.28 ± 48.94	28.85 ± 18.21	197.19 ± 51.68	142.39 ± 24.53	29.31 ± 5.71
	2022	163.83 ± 1.57	24.54 ± 13.61	276.42 ± 192.16	199.53 ± 60.66	27.58 ± 17.44
‘Bastardo’	2021	57.85 ± 22.39	12.48 ± 3.36	93.18 ± 42.51	64.76 ± 18.53	15.77 ± 2.24
	2022	64.17 ± 21.70	14.56 ± 1.47	138.62 ± 30.71	68.95 ± 2.57	27.35 ± 10.38
‘Cabernet Sauvignon’	2021	308.75 ± 15.39	48.71 ± 20.43	296.65 ± 22.73 *	235.98 ± 64.59	68.65 ± 19.53 *
	2022	314.92 ± 119.67	67.85 ± 22.44	527.84 ± 204.83 *	240.76 ± 78.26	125.01 ± 56.50 *
‘Casculho’	2021	200.69 ± 99.49 **	17.34 ± 7.88	224.85 ± 96.78 *	182.13 ± 61.34	43.21 ± 21.11 *
	2022	412.30 ± 21.30 **	34.38 ± 9.85	482.69 ± 168.90 *	271.66 ± 64.42	88.13 ± 25.49 *
‘Castelão’	2021	131.82 ± 44.35	34.39 ± 27.25	234.88 ± 84.72	182.60 ± 71.02	30.54 ± 15.23
	2022	172.35 ± 8.21	33.33 ± 10.13	248.80 ± 65.74	245.41 ± 15.25	32.36 ± 0.78
‘Cornifesto’	2021	105.59 ± 37.40	14.96 ± 3.57	116.56 ± 66.04	40.44 ± 13.38 **	21.08 ± 12.46
	2022	162.84 ± 21.80	24.82 ± 7.90	259.86 ± 118.73	213.22 ± 77.30 **	46.50 ± 27.13
‘Donzelinho Tinto’	2021	127.82 ± 64.27	26.19 ± 11.25 *	535.50 ± 251.60	134.05 ± 38.31 ***	32.98 ± 14.21
	2022	197.88 ± 12.72	52.46 ± 10.68 *	672.61 ± 91.08	492.65 ± 123.50 ***	70.38 ± 66.05
‘Malvasia Preta’	2021	469.53 ± 23.73 **	31.42 ± 7.08	416.99 ± 78.91	223.34 ± 78.43	48.86 ± 0.02
	2022	282.33 ± 23.91 **	24.58 ± 0.12	262.38 ± 126.96	310.88 ± 126.89	37.63 ± 8.09
‘Marufo’	2021	138.67 ± 44.64	29.72 ± 5.88	305.07 ± 130.92	97.42 ± 32.20	36.17 ± 16.82
	2022	113.69 ± 50.04	28.64 ± 14.59	337.83 ± 224.31	128.28 ± 38.37	51.70 ± 35.03
‘Mourisco-de-Semente’	2021	189.47 ± 66.70	50.96 ± 23.01	264.47 ± 149.66	177.38 ± 48.99	58.79 ± 17.99
	2022	210.47 ± 45.03	27.98 ± 10.10	367.47 ± 57.32	268.52 ± 132.57	76.33 ± 23.42
‘Rufete’	2021	154.47 ± 54.66	18.96 ± 5.12	110.45 ± 61.34	111.89 ± 58.28	LOQ
	2022	219.04 ± 37.64	15.27 ± 3.32	147.10 ± 66.60	218.39 ± 106.71	33.46 ± 18.38
‘Syrah’	2021	155.86 ± 26.26	26.95 ± 10.39	108.50 ± 27.36	159.33 ± 58.28	27.16 ± 13.67
	2022	169.91 ± 21.94	37.42 ± 15.02	155.23 ± 38.57	203.49 ± 46.18	29.86 ± 2.63
‘Tinta Barroca’	2021	195.32 ± 33.16	50.11 ± 10.00	238.45 ± 42.68 *	68.11 ± 9.17	72.13 ± 11.10 **
	2022	299.75 ± 140.44	42.06 ± 12.03	451.31 ± 284.31 *	115.63 ± 68.33	145.86 ± 53.72 **
‘Tinta Caiada’	2021	366.15 ± 172.69	30.79 ± 10.87	259.95 ± 137.02	138.58 ± 22.97 ***	LOQ *
	2022	485.34 ± 68.17	34.82 ± 4.28	462.41 ± 35.31	449.73 ± 143.29 ***	56.27 ± 10.40 *
‘Tinta Carvalha’	2021	109.48 ± 21.16	38.34 ± 4.74 *	530.59 ± 105.65 *	89.65 ± 41.77 *	60.84 ± 18.30
	2022	179.46 ± 89.38	63.39 ± 22.96 *	790.51 ± 110.72 *	251.74 ± 94.42 *	64.33 ± 36.92
‘Tinta da Barca’	2021	204.60 ± 66.58	54.23 ± 19.79	231.06 ± 138.97	161.96 ± 64.42	58.86 ± 14.72
	2022	263.98 ± 92.61	64.32 ± 31.98	360.71 ± 118.85	271.19 ± 138.19	69.09 ± 9.08
‘Tinta Francisca’	2021	221.84 ± 33.78	15.88 ± 5.97	169.64 ± 97.85	58.90 ± 32.82	44.63 ± 13.03
	2022	246.00 ± 81.35	21.65 ± 0.94	296.85 ± 30.50	155.68 ± 81.53	42.25 ± 27.82
‘Tinto Cão’	2021	172.57 ± 22.72	48.98 ± 2.18 *	57.05 ± 14.17 *	102.95 ± 20.67 ***	23.46 ± 3.79
	2022	256.98 ± 88.66	22.51 ± 8.54 *	296.89 ± 153.18 *	319.20 ± 177.15 ***	58.41 ± 29.91
‘Touriga Fêmea’	2021	215.50 ± 67.66	16.55 ± 6.36	277.62 ± 84.38	117.23 ± 62.83 **	26.16 ± 12.86
	2022	215.29 ± 38.60	30.08 ± 6.34	386.73 ± 123.68	287.75 ± 94.52 **	32.09 ± 13.72
‘Touriga Franca’	2021	295.62 ± 34.08	71.90 ± 15.46 **	337.09 ± 62.78	244.07 ± 119.12 *	82.69 ± 15.05
	2022	326.05 ± 170.86	36.04 ± 15.76 **	352.38 ± 97.30	391.23 ± 156.06 *	54.87 ± 20.88
‘Touriga Nacional’	2021	353.46 ± 135.34	30.11 ± 5.18	343.11 ± 72.42	200.62 ± 47.18	50.44 ± 18.44
	2022	289.53 ± 46.52	21.96 ± 5.73	265.72 ± 35.45	233.24 ± 57.80	60.6 ± 10.72
‘Trincadeira’	2021	142.11 ± 40.81	15.13 ± 0.47	101.16 ± 59.41	95.51 ± 35.45	28.47 ± 2.95
	2022	199.22 ± 55.56	LOQ	99.71 ± 27.76	204.93 ± 108.60	LOQ
‘Vinhão’	2021	564.79 ± 71.32 ***	88.51 ± 24.80 ***	134.32 ± 59.32	207.14 ± 54.46	28.78 ± 5.59
	2022	341.75 ± 89.91 ***	24.30 ± 2.08 ***	219.80 ± 66.61	226.70 ± 108.32	27.32 ± 1.06
‘Zinfandel’	2021	358.32 ± 87.79 ***	40.56 ± 14.63	590.90 ± 223.65	231.44 ± 97.59 *	184.9 ± 35.14
	2022	586.41 ± 238.93 ***	44.30 ± 20.26	709.51 ± 330.16	390.57 ± 68.59 *	219.54 ± 89.67
*p*(V)		<0.001	<0.001	<0.001	<0.001	<0.001
*p*(Y)		<0.001	n.s.	<0.001	<0.001	<0.001
*p*(V × Y)		<0.001	<0.001	n.s.	n.s.	n.s.

## Data Availability

The original contributions presented in this study are included in the article. Further inquiries can be directed to the corresponding author.

## Abbreviations

The following abbreviations are used in this manuscript:

ABTS•^+^	2,2′-azino-bis (3-ethylbenzothiazoline-6-sulphonic acid)
ANOVA	Analysis of variance
CE	Catechin equivalents
DPPH	2,2-Diphenyl-1-picrylhydrazyl
DW	Dry weight
FRAP	Ferric Reducing Antioxidant Power
GAE	Gallic acid equivalents
HPLC-DAD	High-performance liquid chromatography with diode array detection
PCA	Principal components analysis
TE	Trolox equivalents
TPC	Total phenolic content
